# Supplementary material for: Patterns of Intron Gain and Loss in Fungi
Source: PLoS Biol. 2004 Nov 30;2(12):e422. doi: 10.1371/journal.pbio.0020422 (PMC532390; doi:10.1371/journal.pbio.0020422)
Supplement: Table S1 — Also available at http://genes.mit.edu/NielsenEtAl/. (4.3 MB ZIP). [file pbio.0020422.st001.zip › NielsenEtAl/html/280.html]

AN1085.1.NCU09610.1.MG06979.1.FG00833.1


```
 CLUSTAL W (1.82) Multiple Sequence Alignments - Introns Inserted


Sequence 1: NCU09610.1	862 aa
Sequence 2: MG06979.1	848 aa
Sequence 3: FG00833.1	859 aa
Sequence 4: AN1085.1	858 aa
Alignment Length: 871 aa
Number Identitical Residues: 490 aa
Alignment Score (without introns) 22945


MG06979.1 	--MNGNFAAVGS------EPTDAQFEHGIQVIDEDKQYN2GLVNDYLRRTHVAEAGFNYH
NCU09610.1	--MNGHFAAVDNGNGSSPDSNAHSFEHGIQVINEDKQYN~TNLNEYLNETHVAEAGFNYH
FG00833.1 	--MNGHFSAVGE------APGAGSYEHGVQVIDEDKEFN2THISEYLQKTNVADSGFNYH
AN1085.1  	MATNGHFAPIGSD-----SSDKTTYEHGVQVIDENKEFN2TNLTKYLTFENVTPAGFNYH
          	 : **:*:.:...      .    :***:***:*:*::*   :..**   :*: :*****

MG06979.1 	LISVFGSQSTGKSTLLNNLFGTEFSVMSESERRQTTKGIWMSKNKREG----KMAENILV
NCU09610.1	LISVFGSQSTGKSTLLNHLFGTQFSVMSERERRQTTKGIWMSKNKNEG----KMADNILV
FG00833.1 	LISVFGSQSTGKSTLLNNLFGTEFSVMSETERRQTTKGIWMSKNKRETSAGTPMSENILV
AN1085.1  	LISVFGSQSTGKSTLLNHLFGTHFSVMAETERRQTTKGIWLSKNKNGD--GKSMADNILV
          	*****************:****.****:* **********:****.    .. *::****

MG06979.1 	MDVEGTDGRERGEDQDFERKSALFALATSEVLIVNIWEHQVGLYQGANMGLLKTVFEVNL
NCU09610.1	MDVEGTDGRERGEDQDFERKSALFALATSEVLIVNIWEHQVGLYQGANMGLLKTVFEVNM
FG00833.1 	MDVEGTDGRERGEDQDFERKSALFALATSEVLIVNIWEHQVGLYQGANMGLLKTVFEVNL
AN1085.1  	MDVEGTDGRERGEDQDFERKSALFALATSEVLIVNIWEHQVGLYQGANMGLLKTVFEVNL
          	***********************************************************:

MG06979.1 	QLFLKDKQ2SSPRSLLFFVIRDHIGNTPLSNLRNTLVQDLTKIWSSISKPPALENAKIED
NCU09610.1	QLFLKDKQ2NQTRSLLFFVIRDHIGVTPLANLRNTLIQDLTHIWSSISKPAGLENSKIED
FG00833.1 	QIFLKDKQ2STPRSLLFFVIRDHLGTTPLGNLRTTLIQDLTKIWSSLSKPQGLEGSRIED
AN1085.1  	QLFLKDKN2TTHRSLLFFVIRDFVGTTPLKALQKTLMEDMSRLWDSISKPPGLERSTVHD
          	*:*****: .  **********.:* ***  *:.**::*::::*.*:*** .** : :.*

MG06979.1 	YFDFAFAALPHKILQPEKFVTEVENLGTRFVAGHR-----STQDQEFVGGVFLPEYHRRI
NCU09610.1	YFDFAFAALPHKILQPDKFISEVQNLGSRFIAGHRNKDSDATDDQELTGGVFLPEYHRRI
FG00833.1 	YFDFGFAALPHKILQADKFTEEVQKLGARFTAGHRHGKAGLHGDQELEGGLFLSEYHRRI
AN1085.1  	YFDFQFYGLPHKSYQPEKFVEETKKLSLRFREGQKNATLNAQNGEFSEGGVFLPEYHRRI
          	**** * .****  *.:**  *.::*. **  *::    .   .:   **:**.******

MG06979.1 	PADGFSIYAEGIWDQIVNNKDLDLPTQQELLAQFRCDEISRDVLNAFDEAITPLEDKQAE
NCU09610.1	PADGLSIYAEGIWDQIVSNKDLDLPTQQELLAQFRCDEIAREVQIAFDAAIAPLEEQQAE
FG00833.1 	PADGFSVYAEGIWDQIVNNKDLDLPTQQELLAQFRCDEIAREVQIDFDVIIAPLEEKQSE
AN1085.1  	PADGFSVYAEGIWDQIVNNKDLDLPTQQELLAQFRCDEILREVMVAFDEAIFPFEDKQSQ
          	****:*:**********.********************* *:*   **  * *:*::*::

MG06979.1 	ASRLGKPFVLPDLGDTARSARSKAVEAFKVQASRYHKGVYTRKQAELEGKMDSRLKALYQ
NCU09610.1	STRAGKPAVLPNLGQIGAEAREKCVKNFETQASRYHKGVYTTKRAELEDKIDNRLKALYQ
FG00833.1 	ASKLGIPTVLPDLGLAGNDARQKCIKAFEVQASRYHKGVYTRKRHELEGKIDTRLKSLYQ
AN1085.1  	ASRLGEPEVLGGLGAAMRSARAKATKNFETEASRYHKGVYQRKRAELEGKVDTRLKALFQ
          	::: * * ** .**    .** *. : *:.:*********  *: ***.*:*.***:*:*

MG06979.1 	GQLAAAHKAGVAAFSDAVTGAVKAGQKSGGSYEFAEIVDKQKRKTLDIFAKEAQGLEIEG
NCU09610.1	AHLTAAHKAGVTAFSEAVANAVKAGQKAGGAYEFAEIVEKQKTKTLEIFKKEAQSLAIPG
FG00833.1 	SQLAAAHKAGVAAFGDAVANKVKAGQKAGGQYEFAEIVANEKRKTLDIFGVEAQSLLIEG
AN1085.1  	GQLNAAHKSGINDFSDAVTAEVKAGQKKGTGYDFAEIVNDEVKKALQKYEEVARATVVEG
          	.:* ****:*:  *.:**:  ****** *  *:***** .:  *:*: :   *:.  : *

MG06979.1 	LAWTNFKPQFLLFEKELDEVSARLRKDEMRRLATRVERWVKSRLGDSIGLEFNKLGSGRG
NCU09610.1	VAWSNFKPQYLIFEKELDEVSARLRKEEMRRLAIRVERWVKSRLGDAIGLEFNKLGSGRG
FG00833.1 	VTWTNFESQLRLFEKELDEESAKLRKEEMRRLATRVERWVRSRLGDAIGLEFNKLGSGRG
AN1085.1  	APWSNYQQELALYEKELSEVSARLRRDEMRRLATRVERWVQSRLGESVGLEFNALGSGRA
          	 .*:*:: :  ::****.* **:**::****** ******:****:::***** *****.

MG06979.1 	GSGAPETGEKPATEKDLWDRIWTVFVAVVKEAQERFAERAKSFEASSEEVEIGLWRLRRK
NCU09610.1	GSGAPESGEKPATEKDIWDRVWKAFISIVGEAESRFTDRAKSFEASDDEVQVGLWRLRRK
FG00833.1 	GSGAPENGEKPETEKDLWDRVWKVFTGIVKEAEVRFAERAKSFDATSEEVEIGSWRLRRK
AN1085.1  	GGAAPESGEKP-SEKKFWDRVWNLFVETVLDAERRFTDRASSFDASLEEVDVGLWRLRRK
          	*..***.**** :**.:***:*. *   * :*: **::**.**:*: :**::* ******

MG06979.1 	SWVALRERIDEEVMEGNILLKLRENFEDKFRYDEAGVPRIWRPTDDIEGIYTRARESTLT
NCU09610.1	SWVALREKIEEEVMESNILMKLRENFEDKFRYDEDGVPRIWRPSDDIEGIYTRARESTLG
FG00833.1 	SWNALREKIEEEVMEGNILLKLRENFEDKFRYDEAGVPRIWRPTDDIEGIYTKARESTLT
AN1085.1  	SWGVLRAKIEEEMTEGNLLLKLRENFEDKFRYDEAGVPRIWRPTDDIEGIYTRARESTLT
          	** .** :*:**: *.*:*:************** ********:********:****** 

MG06979.1 	LIPLLSRFRLAETYASPDLPGWIGNQPPGVEPDDEEDLTPIGGIDEEEGKSLEEEMTVLS
NCU09610.1	LVPLLSRFRLTSTSAPPDLIEFVGPQPHGVEPGDEEDLTPIGGVDEDEGKSLEEETTILS
FG00833.1 	LVPLLSRFRLSETYGPPDLPGFVGAQPSGVEASDEEDLAPIGGIDEEDGKSLEEEMTVLS
AN1085.1  	LIPLLSRFRLAETSAPPPLDRWVGHTPSSATTADEEDLPPIGGVDEEEGKSLEEEMTILG
          	*:********:.* ..* *  ::*  * .. . *****.****:**::******* *:*.

MG06979.1 	ESKRQDLVVRFKKTADGVYVEAKRGALGGMTQVPLYFWIALFAFGWNEIWMV1IRNPFLF
NCU09610.1	EPKKQDLVVRFKKMADGVYVEAKRSAIGGITQVPLYFYAVLLVLGWNEFVMV1LRNPILF
FG00833.1 	EGKRQDLVVRFKKTADGVYVEAKRGAIGGVAQIPWYFYGLLLALGWNEILTV1LRNPFLC
AN1085.1  	DAKRQELTIRFKKAADGVYVEAKRSAIGGMTQVPLYFYGILLALGWNEIVAV1LRNPAYF
          	: *:*:*.:**** **********.*:**::*:* **:  *:.:****:  * :***   

MG06979.1 	ILLLLSAGGTYVAYNLSLLGPMMQMTNAAANQASEIGKQKLREFLENNETARQALAMPAS
NCU09610.1	LLLLLISGGTYVAYSLNLLGPMMQMANAASTQGMEIGKAKLRDFLENHEGARGALGMPPK
FG00833.1 	LLILVMAGGTYLAYTLNLLGPMLSMSNAAMTQGVEIAKQQLRDFIANSDTARQAVGMPAR
AN1085.1  	FLLFVCAVGAYVTYQLNLWGPILKMTEAASNQAMIEGKRRLREFLETSDTGRQAIAMSSS
          	:*::: : *:*::* *.* **::.*::** .*.   .* :**:*: . : .* *:.*.. 

MG06979.1 	SKSSGGEQVRMDTLDSKGKKKDY---DDDGI---
NCU09610.1	NAQRVESGISMDTLDSNGKRKETGLGAEDDI---
FG00833.1 	QDS---DNISMDTLDSRGKKANS-TAEKDDIDDI
AN1085.1  	GSSRSGNEHEMSRLNKQGKSSTD--EDVDDL---
          	  .   .   *. *:..**         *.:
```
